# Bridging Connectivity Issues in Digital Access and Literacy: Reflections on Empowering Vulnerable Older Adults in Singapore

**DOI:** 10.2196/34764

**Published:** 2022-05-03

**Authors:** Haikel A Lim, Joanne Sze Win Lee, Meng Han Lim, Lynn Pei Zhen Teo, Natalene Siew Wen Sin, Rou Wei Lim, Si Min Chua, Jia Qi Yeo, Nerice Heng Wen Ngiam, Angeline Jie-Yin Tey, Celine Yi Xin Tham, Kennedy Yao Yi Ng, Lian Leng Low, Kai Wen Aaron Tang

**Affiliations:** 1 Department of Psychiatry National Healthcare Group Singapore Singapore; 2 TriGen Singapore Singapore; 3 Duke-NUS Medical School Singapore Singapore; 4 Population Health and Integrated Care Office Singapore General Hospital Singapore Singapore; 5 National University of Singapore Singapore Singapore; 6 Division of Pharmacy Singapore General Hospital Singapore Singapore; 7 Punggol Polyclinic Singapore Health Services Singapore Singapore; 8 National Healthcare Group Pharmacy Singapore Singapore; 9 Department of Respiratory Medicine Singapore General Hospital Singapore Singapore; 10 Division of Medical Oncology National Cancer Centre Singapore Singapore; 11 Department of Family Medicine and Continuing Care Singapore General Hospital Singapore Singapore; 12 Department of Psychiatry Singapore General Hospital Singapore Singapore

**Keywords:** COVID-19, digital literacy, digital literacy training, digital disparities, digital divide, social construction of health technologies, health technology, COVID-19 pandemic, pandemic, COVID, social isolation, elder, older adult, Asia, access, barrier, empower, volunteer, vulnerable, digital skill, low income

## Abstract

This article describes a ground-up initiative for a volunteer-run digital literacy program in Singapore targeting vulnerable older adults, focusing on the barriers faced in running this program and training these beneficiaries. It further offers possible solutions to overcome these hurdles, providing insight for individuals or organizations seeking to start similar ground-up initiatives.

## Introduction

Digitalization is a phenomenon that has become increasingly prominent over the years, as countries seek to adapt to the changing world and improve the standards of living of their citizens [1]. Singapore, an island state in southeast Asia, has not been spared from this digital wave, and it has developed plans to facilitate digitalization from as early as the 1980s [2]. Although digitalization is often quoted as a boon to many, there remain those who have not been recipients of its bounty [3]; sociodigital divides are surfacing between generations [4], with vulnerable older adults increasingly being unable to keep up with such rapid digital progress [5].

On January 23, 2020, the first case of COVID-19—a coronavirus that sparked a global pandemic in 2019—touched the shores of Singapore [6]. As with other international societies, swift lockdown measures were put in place to limit the spread of the virus [7]. Such measures limited the physical interaction of day-to-day activities, leading to companies and organizations shifting their businesses to internet-based platforms. Digital literacy thus became essential to run even the most basic of errands, further exacerbating and exposing the pre-existing inequalities of the digital divide [8]; technologically illiterate older persons [9], already a vulnerable subset of the population [10], not only lost their access to essential services and the community [11] but also their quality of life and a sense of well-being [12].

Different groups of volunteers in Singapore then collaborated during the global COVID-19 pandemic and sought to improve the digital access and literacy levels of these now socially isolated older adults. However, the efforts to reach vulnerable older adults (those older than 60 years) in Singapore were fraught with difficulties and challenges, and these were made possible only through various interventions at the societal, grassroots, and individual levels. In this article, a ground-up initiative for a volunteer-run digital literacy program in Singapore targeting vulnerable older adults (Project Wire Up) is briefly described; reflections from the planning and execution of this initiative are then divided into (1) barriers faced and the individual, grassroots, and societal interventions that helped facilitate digital literacy in this vulnerable population; and (2) possible solutions to overcoming these hurdles, providing insight for individuals or organizations seeking to start similar ground-up initiatives. Considering the pandemic, this initiative was only started when in-person visits by hospital volunteers were allowed, in accordance with existing government regulations during the lockdown period.

## Project Wire Up

### Description

Project Wire Up is an ongoing ground-up initiative by volunteers from TriGen Ltd, a nonprofit organization based in Singapore, in collaboration with the Singapore General Hospital [13]. Project Wire Up started in 2020 and aims to address social isolation and a lack of access to essential services among older adults by improving digital access and literacy levels. In particular, this program targets vulnerable and socially isolated older adults of a lower socioeconomic status (ie, those living in rental public housing apartments or those receiving financial assistance). As of the end of 2021, more than 300 of these vulnerable older adults have benefited from the program, with new participants being enrolled and trained every day.

The program adopts a 3-pronged approach where older adults are (1) equipped with smartphones, (2) trained by volunteers for 6 sessions over 3 months, and (3) reconnected to their social networks. To equip these older adult participants with the tools to facilitate digital literacy, special arrangements were made with local telecommunication companies and the Infocomm Media Development Authority of Singapore (IMDA), a statutory board in Singapore that develops and regulates the information communications and media sectors of Singapore [14], to offer subsidized smartphones as well as mobile network and data plans to these participants. Prior to the availability of these schemes, the program relied on goodwill donations of smartphones from the public together with promotional “limited-time-only” mobile plan schemes from local telecommunication companies.

Digital skill training is facilitated by trained volunteers, mainly in health care–related fields, matched with these participants based on language and location. Volunteers guide participants through a tiered curriculum of increasing difficulty that is personalized according to the needs, wants, and abilities of the participants. The curriculum often starts with empowering these participants to carry out basic hardware operations, including essential functions such as making phone calls and establishing contact lists. Following this, participants are then taken through a tiered curriculum that involves educating the participants on (1) communication platforms (eg, WhatsApp, Telegram), (2) government services and lifestyle apps, and (3) electronic payment and digital banking. Table 1 presents the details of the curriculum [15,16]. Participants are then further educated regarding the importance of cybersecurity and identification of scams (including phishing) to ensure their digital safety.

**Table 1:** Summary of the tiered curriculum [16].

Tier number	Tier name	Examples of topics taught
0	Basic operation of hardware	Turning the phone on and off, charging the phone, adding to and searching through contact list, sending text messages, and making calls
0	Cybersecurity and scams	How to spot a scam, staying safe when on the internet, protecting personal data, and securing accounts on media platforms
1	Communication skills and platforms	Email, Google, WhatsApp, Telegram, Zoom, Microsoft Teams, Facebook, Instagram, and Tiktok
2	Government services and lifestyle apps	YouTube, local entertainment apps, local government service apps, getting around (eg, Grab, local taxi services), and local health apps
3	Electronic payments and digital banking	Local bank, local supermarket, and food delivery apps

### Ethics Approval

As this paper only describes a volunteer-based initiative in Singapore, no ethics approval was required.

## Barriers to Improving Digital Access and Literacy in Vulnerable Older Adults

Commencing a ground-up volunteer-based initiative lends itself to various difficulties, and Project Wire Up is no exception. The barriers faced when facilitating digital literacy and access among vulnerable older adults are described below, ranging from more macrolevel issues, such as acquiring funding for the program, to more microlevel issues, such as being able to engage the elderly. These barriers are grouped into the following categories: financial and logistical barriers, establishing initial contact and engaging the elderly, and cognitive impediments.

### Financial and Logistical Barriers

To effectively execute a program aimed at empowering older adults with smartphones and the knowledge to use them requires the availability of the following basic resources: smartphones and the mobile or data plans. One of the concerns brought up most commonly by participants as a barrier to them having previously accessed this resource is the cost involved in purchasing new or used mobile devices and subscribing for the appropriate mobile phone plans. This proved to be a significant concern given that participants were already from a lower socioeconomic stratum, which would preclude them from accessing financial resources to make such purchases. Although the physical devices themselves could be obtained through donation drives or through a 1-time purchase using donations in cash, it was challenging to identify a reliable, realistic, and sustainable way to support these participants in financing their own mobile phone plans.

Mobile phone plans in Singapore are either prepaid, in which a sum of money is paid to the mobile phone plan service provider and deductions are made from this predetermined amount in the account based on the usage, or postpaid, in which the individual pays at the end of the month based on the usage and costs incurred during the preceding month. As such, regardless of the type of payment plan, using a smartphone or mobile device would inadvertently incur a recurring cost over a long period. Special requests to local telecommunication companies to indefinitely extend advertised “promotional limited-time-only” deals to Project Wire Up beneficiaries were for naught, understandably, given (1) hesitancies regarding the risk of defaulting payments from the program beneficiaries and (2) logistic requirements at repeatedly issuing these deals only for these specific individuals and tracking them over time. Therefore, the sustainability of any improvements made for improving digital access among this population was under threat.

Initial alternative solutions considered for financing these plans were also problematic. Under the Singapore law, individuals are not allowed to purchase telephone connections for others, given the responsibility to be undertaken for any activities, illegal or otherwise, done via telephone; therefore, the program and telecommunication companies were unable to make such a compromise. Although the participants themselves might be able to afford the initial set-up and purchase, it was logistically unfeasible for charitable monetary donations to be channeled for making monthly depositions or payments either into the beneficiaries’ mobile phone accounts or even to the beneficiaries, owing to concerns regarding whether that money would truly be used for this purpose.

### Difficulties in Engagement

Singapore is a multiethnic country with the lingua franca generally being Malay (Bahasa Melayu Singapura) among the older adults (although now only spoken by an ethnic minority of younger persons in Singapore), and Singapore Standard English among the younger adults and youth [17]. A significant number of these older adults were not English educated and hence were most comfortable in speaking their dialects (as opposed to Singapore Standard Mandarin or Bahasa Melayu Singapura) [18]. As with all heterogenous societies, language proved to be a significant barrier for volunteers that had to be overcome; however, before even such language barriers could be overcome, commencing sessions with these older participants involved establishing initial contact, and this proved to be a significant barrier to facilitating digital literacy and access.

Initial contact was made primarily through telecommunication; however, with the rising number of scams in Singapore [19], these older participants were wary of volunteers. As such, building trust and rapport via telecommunication or in person understandably took a while. This was further confounded by individual characteristics; some participants, despite their desire to learn, were by personality reticent or slow to warm up, and allowing strangers into their personal space took a significant amount of time, trust, and rapport. In some cases, after a few visits for a multitude of reasons, participants declined volunteer visits and were lost to follow-up.

Furthermore, as the program continued, it was noted that these older adults differed considerably in their abilities to pick up new digital skills, partly due to their different educational backgrounds and physical conditions. As expected with any training program, participants had varying levels of interest as well as different needs and wants that had be addressed. As such, despite the tiered “formal” curriculum, this program ended up serving primarily as a guide, using which each volunteer needed to review and identify aspects that were relevant and personalized to their beneficiaries.

### Physical and Cognitive Impediments

As part of eventual government-directed funding for this program, it was compulsory for participants to attend digital learning programs at learning hubs distributed at various locations in the country and pick up at least 1 basic digital skill before they could qualify for this program. Although the requirement that older adults had to pick up at least 1 basic digital skill at specific locations was well intended, this disadvantaged many older adults who often have physical impediments.

Thus, these participants with physical impairments were also less likely to engage in the acute uptake of technology, as documented clearly in earlier studies [20]. For example, some older adults had visual impediments that prevented them from clearly viewing the icons or buttons on the mobile device. Others had underlying dexterity issues, possibly secondary to neuropathy, resulting in challenges when navigating the newer mobile devices that are typically operated using touch screens instead of mechanical buttons.

Furthermore, given the target population, a significant number of the beneficiaries of this program had suffered from various forms of subtle and sometimes explicit cognitive impairment, either formal or subclinical. Such impairments have been known to impede the uptake and usage of digital technology [20]. Participants often could not recall volunteers’ multiple visits, much less the complex steps to achieve an outcome on the smartphone. Repeated visits for the same lesson plan were often required before older adults were deemed to have mastered that particular skill set (sometimes involving rudimentary skill sets such as identifying the application of a phone to even make outgoing calls).

### Ageist Attitudes

Of particular note is also the concept of self-ageist attitudes, which might exacerbate all the preceding barriers, with the elderly themselves sometimes imbibing and reinforcing such stereotypes [21]. Volunteers highlighted that self-deprecating stereotypes about their age from these older adults themselves significantly contributed to participants giving up easily in view of the multiple intermediate steps often needed to access certain services. Many participants also expressed a fear of technology related to their anxiety with digital devices, consistent with local findings suggestive of this phenomenon [22].

## Overcoming Barriers to Improving Digital Access and Literacy in Vulnerable Older Adults

There are several barriers to achieving digital access and literacy; although a majority of these are often within the locus of control of individuals, many of these barriers require interventions at the societal and grassroots level. The following section details the micro- and macrolevel interventions and assistance that helped overcome these barriers to digital access and literacy among a vulnerable group of older adults.

### Government and Societal Level

#### Government Support in Lowering Operational Barriers to Digitalization

The initial phases of the program relied entirely on mobile phones donated by the public and time-limited goodwill extensions of promotional deals for mobile phone plans from telecommunication companies. Thankfully, a few months into the pandemic, the Singapore government launched a subsidized mobile device scheme for underprivileged older adults as part of the larger “Seniors Go Digital” initiative [15]. This scheme provided subsidized smartphone and mobile plans to financially deserving older adults (aged 60 and above) who were keen on embracing digital technology but could not afford it. This ensured that the financial and logistic barriers to the sustainability of this program were largely removed.

### Grassroots and Community Level

#### Reaching Vulnerable Older Adults Through Grassroots Organizations

Grassroots organizations played a significant part in ensuring the success of this digital access and literacy program. First, these organizations facilitated the identification of at-risk vulnerable adults who might be open to embracing digitalism, capitalizing on their knowledge of these vulnerable older adults residing in their vicinity. Second, as these older adults themselves were also familiar with the staff of these grassroots organizations, the support of these organizations allayed suspicions toward Project Wire Up volunteers, which have been heightened considering the recent local prevalence of scams [19]. As such, to facilitate participation and enrollment, staff from the grassroots organizations would aid the team in making the first visit to potential participants to inform them of the program prior to first contact from program volunteers.

#### Facilitating the Last Mile Delivery

Although governmental efforts may reduce operational barriers to obtaining and paying for a mobile phone and plan, they often come with a caveat and requirement. In case of the “Seniors Go Digital” initiative, participants who had expressed interest in the program found themselves hampered by the last mile delivery of phones and setting up of digital plans. In particular, potential participants had difficulties in going to learning hubs to learn digital skills or going to distribution hubs to collect their phones due to physical limitations and the general inconvenience in doing so, especially with concerns related to the ongoing pandemic. Project Wire Up closed this gap by engaging with digital telecommunication companies, with the support of the IMDA [14], to bring the equipping and training process to the participants themselves via door-to-door outreach efforts.

Prior to a scheduled outreach program, a list of interested older adults would be prepared with assistance from grassroot organizations via door-to-door visits in the neighborhood and they would be invited to attend the event. Thereafter, on the day of the outreach program, telecommunication companies would bring their staff and the digital devices to a location in the neighborhood to aid in the registration process. Volunteers would bring these potential participants to the venue of the outreach program and thereafter accompany them back to their respective homes to commence the training sessions.

#### Linking Older Adults to a Digital Community

It has been well demonstrated that older adults who are more socially connected are more likely to use digital technology [23]. It naturally follows that the sustainability of this continued use of digital devices was also partly dependent on whether these vulnerable older adults were eventually linked, or connected, to a digital community. Based on the experience of this program, this connection often occurred in the form of WhatsApp or Telegram groups with neighbors and volunteers or periodic “activities over Zoom” organized by volunteers and grassroots organizations. For example, a grassroots organization facilitated the implementation of the “Radin Mas Silver Click!” program, a recurrent monthly program involving health talks, exercise classes, cooking classes, and support groups over Zoom in which any elderly adults in their catchment area or community could participate once they were equipped with the appropriate digital devices. Although not all individuals may be keen on joining digital communities and virtual event platforms, with various factors potentially influencing their willingness to join digital communities [24], providing the option for those who are keen is the first step toward facilitating sustained and perhaps even improved digital literacy and access.

### Individual or Volunteer Level

#### Motivating Older Adults

Communicating with participants in their preferred spoken language or mother tongue (dialect) proved helpful; however, despite language barriers, sessions were more fruitful when they were not too result-oriented and when volunteers themselves focused on genuinely helping the participants develop their digital literacy skills. On doing so, participants were more willing to proceed at a pace they were comfortable with, which further motivated them to continue learning (as opposed to finding the endeavor highly insurmountable).

Targeting and personalizing the training, especially at the beginning, to the participants’ lifestyles and perceived needs were helpful in ensuring participants’ investment in the initial phase of the learning journey. This finding corroborates existing ones suggesting that a key enabler in digital literacy programs is helping older adults see the relevance of and need for digital technology [25]. For example, those who were avid television or music connoisseurs were shown the capabilities of video and music streaming apps; identifying particular interests (cooking, news, art, jazz, etc) also further engaged participants and motivated them to continually learn about the app functions. In tandem with this, engaging caregivers or family members of these older adults and focusing on social connectivity with friends further encouraged participants to be proficient in apps such as video calling.

Volunteers in this program also noted that as they gradually got to know their participants and developed an amicable relationship with them over time with their repeated visits, these older adult participants were gradually more receptive to their suggestions, and they were also more willing to try learning new skills [26]. This goes in tandem with recent geragogy studies in this region, where ageist stereotypes could often be countered by relatable, empathetic, and engaging instructors [27].

#### Teaching Simply and Using a Tiered Curriculum

Pacing the teaching and repeating sessions, although often onerous for the volunteers and participants, showed greater results. Older adults found themselves able to absorb new knowledge when complex steps were simplified into algorithms that were easier to understand. Repeated sessions facilitated rapport building, which in turn allowed participants to progress at their own pace. By prioritizing functions, participants who were not proficient at the get-go were found to have progressed better if simple functions including turning the phone on and off, unlocking the phone, charging the phone, and even accepting or rejecting calls or entering contacts and clicking photographs were introduced first. In doing so, with repeated reassurance, coaxing, and encouragement, more difficult features such as electronic payments and internet banking, video streaming, and even ride-hailing apps could be introduced and taught.

The usage of a tiered curriculum (given in Table 1) [16] facilitated this process; volunteers had an idea of what skills were “easier” or “harder,” and these provided a roadmap for volunteers regarding which groups of digital skills they could teach their beneficiaries. This allowed for a more progressive pacing of the training. This efficacy of using a flexible curriculum and having personalized curriculums tailored to the learner’s capabilities is also consistent with recent geragogy studies in the region [27].

#### Using Aide-mémoire

Given the complex nature of learning, volunteers found that older adults who were more successful were diligent with taking notes or writing and recording the steps. Volunteers further facilitated this process by writing things down, typing them on a phone pad, or capturing a photograph of the written instructions. For those who were illiterate, voice and audio recordings with instructions from volunteers were also helpful. Furthermore, within reason, volunteers were available for assistance if participants contacted them outside of these sessions. Certain smartphone models also allowed for the activation of accessibility options like readback for visually impaired participants, which significantly improved their usage of the smart devices.

Giving participants homework also facilitated learning, allowing volunteers track the participants’ progress; this also involved random “tests” during which participants were contacted by volunteers and asked to assist in certain tasks. Unfortunately, for participants who were more passive learners, such a learning style was unsuccessful; therefore, volunteers spent a significant amount of time finding out what worked best for each participant.

#### Using Accessibility Functions on Mobile Devices

Given that many older adults have physical impediments, volunteers found various solutions for the different physical impediments that the older adults may have. For example, for older adults with visual impairments, volunteers increased the font size, contrast, and brightness of the device. Alternately, some volunteers used devices with larger screens. In addition, volunteers taught older adults how to use the voice-enabled functions in the phones (eg, Siri for Apple iPhones or voice-recorded messages in the WhatsApp messaging app). Despite this, volunteers found that given the linguistic issues or preferred language of communication, many of these voice-assisted devices do not recognize dialects [28], which are often the predominantly spoken languages among older Chinese adults of lower socioeconomic status in Singapore aged 55 and older [29].

For older adults with dexterity issues, the touch screens were made less sensitive. However, some older adults still found it challenging to navigate a touch screen and ended up being “demoted” to use an older device employing mechanical buttons. It became clear that though volunteers attempted to help older adults overcome these physical impediments, there still exist systemic gaps in the mobile device industry to meet the needs of older adults, especially those who are illiterate and suffer from various physical impediments. More efforts are required from the private and public sectors to provide high-quality mobile devices that can help older adults, especially those with low socioeconomic statuses, overcome their physical impediments.

### Conclusions

This pandemic era has shown us the importance of embracing digitalization and how the elderly population has struggled to keep up with this changing tide. Although volunteer-based ground-up initiatives are important in helping this population, they face several difficulties. This article highlights some barriers that similar programs might face in facilitating digital access and literacy among their participants, and we hope that the lessons we have shared may be of value in the development of other similar volunteer-based ground-up initiatives in other parts of the world.

## Abbreviations

IMDA: Infocomm Media Development Authority of Singapore
